# Nuclear Microautophagy Drives Vacuolar Targeting of Yeast Iron‐Regulated Proteins During Lipid and Iron Limitation

**DOI:** 10.1002/mbo3.70278

**Published:** 2026-04-16

**Authors:** Tania Jordá, Sergi Puig

**Affiliations:** ^1^ Departamento de Biotecnología, Instituto de Agroquímica y Tecnología de Alimentos (IATA) Consejo Superior de Investigaciones Científicas (CSIC) Paterna Valencia Spain

**Keywords:** Aft1, autophagy, Cth2, iron deficiency, unsaturated fatty acids, yeast

## Abstract

Iron is an essential cofactor involved in cellular processes, including energy generation and the biosynthesis of DNA, proteins, and lipids. The limited solubility of iron at physiological pH frequently results in iron deficiency, thus necessitating sophisticated regulatory mechanisms to maintain iron homeostasis. In *Saccharomyces cerevisiae*, the transcription factor Aft1 mediates the early response to iron limitation by accumulating in the nucleus and activating the iron regulon, a set of genes involved in iron uptake, utilization and sparing. One of Aft1 targets, *CTH2*, encodes for a protein that promotes iron economy by post‐transcriptionally downregulating non‐essential iron‐dependent pathways. Yeast cells that exhibit defects in unsaturated fatty acid (UFA) biosynthesis, such as *mga2Δ* mutants, mislocalize Aft1 to the vacuole under iron‐deficient conditions, which impairs activation of the iron regulon. In this study, we show that Cth2, but not other nucleo‐cytoplasmic shuttling proteins, also accumulates in the vacuole under simultaneous UFA and iron deficiencies. The deletion of autophagy‐ and piecemeal microautophagy of the nucleus (PMN)‐related genes, including *ATG1* and *NVJ1*, prevents Aft1 vacuolar mislocalization. Furthermore, the subcellular distribution of Nvj1 supports PMN activation under these conditions. Despite preventing vacuolar accumulation, these mutations do not restore the regulatory functions of Aft1 and Cth2, nor do they rescue growth in low‐iron conditions. These findings suggest that PMN selectively targets non‐functional iron‐regulated proteins for degradation when both iron and UFA levels are limiting, serving as a quality control mechanism rather than a pathway for functional recovery. These findings underscore a regulatory layer coordinating nutrient sensing and protein turnover.

## Graphical abstract

Aft1 and Cth2 are essential for *Saccharomyces cerevisiae* adaptation to iron deficiency, but in *mga2Δ* cells, PMN drives their mislocalization to the vacuole. Although PMN inhibition reduces this mislocalization, it does not restore their functional activity. Created in BioRender. Puig, S. (2026) https://BioRender.com/gfhuez3.

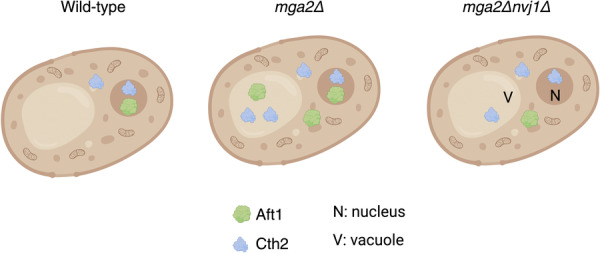

## 1 Introduction

Iron is an essential micronutrient for all eukaryotic organisms. Its redox properties enable its participation as a cofactor in many cellular processes, including energy generation, and DNA, protein, and lipid biosynthesis. Ferric iron is highly insoluble at physiological pH, frequently leading to nutritional iron deficiency, which causes iron deficiency anemia and ferric chlorosis in animals and plants, respectively (Briat et al. 2015; Means 2020). Living organisms have developed intricated regulatory mechanisms to maintain iron homeostasis (Dutt et al. 2022; Riaz and Guerinot 2021). The budding yeast *Saccharomyces cerevisiae* has been used as a model eukaryotic organism to understand the molecular basis that governs iron metabolism (De Freitas et al. 2003; Ramos‐Alonso et al. 2020).

In response to iron deficiency, the nucleo‐cytoplasmic shuttling and iron‐sensing transcriptional factor Aft1 of *S. cerevisiae* accumulates in the nucleus and activates the transcription of a set of genes known as the iron regulon (Ueta et al. 2012; Yamaguchi‐Iwai et al. 2002). This group of genes includes proteins that encode: (i) the multicopper ferroxidase Fet3 and the iron permease Ftr1, which together form the high‐affinity iron uptake system, (ii) cell wall proteins such as Fit3, which enhance iron acquisition, and (iii) the mRNA‐binding protein Cth2, which economizes iron by prioritizing its utilization in essential over dispensable iron‐dependent processes (Rutherford et al. 2003; Shakoury‐Elizeh et al. 2004). Cth2 is a nucleo‐cytoplasmic protein that binds in the nucleus to mRNAs containing AU‐rich elements (AREs) within their 3'‐untranslated region (3'‐UTR) (Perea‐Garcia et al. 2020; Vergara et al. 2011). Upon binding to RNA, Cth2 is exported to the cytoplasm, where it limits the expression of its target mRNAs by inhibiting their translation and promoting their degradation (Pedro‐Segura et al. 2008; Perea‐Garcia et al. 2020; Puig et al. 2005; Puig et al. 2008; Ramos‐Alonso et al. 2020; Vergara et al. 2011). In response to iron deficiency, Cth2 preferentially targets transcripts encoding proteins that participate in the mitochondrial electron transport chain, such as those encoding subunits of the succinate dehydrogenase complex (e.g., *SDH2* and *SDH4*), to limit iron utilization in mitochondrial respiration (Perea‐Garcia et al. 2020; Puig et al. 2005; Puig et al. 2008; Romero et al. 2019).

Multiple lines of evidence have established a close relationship between iron and lipid metabolism. In yeast, the biosynthesis of unsaturated fatty acids (UFAs) requires the participation of the iron‐dependent enzyme Δ9 fatty acid desaturase Ole1 (Stukey et al. 1989). Consequently, in response to iron limitation, the activity of this enzyme diminishes and the levels of UFAs decrease (Shakoury‐Elizeh et al. 2010). As a result, yeast cells activate the expression of *OLE1* when iron is scarce through the Mga2 transcription factor (Romero et al. 2018). Indeed, *mga2Δ* mutants exhibit defects in UFA biosynthesis, especially in iron‐depleted conditions (Romero et al. 2018). Remarkably, *mga2Δ* mutants display a severe growth defect in iron deficient conditions, which can be reverted by supplementing the growth medium with UFAs or by expressing *OLE1* under the control of an Mga2‐independent promoter (Romero et al. 2018). *mga2Δ* cells exhibit a defect in the transcriptional activation of the iron regulon that can be partially rescued by the over‐expression of a constitutively active Aft1 transcription factor (Jorda et al. 2020) Subcellular localization experiments have revealed that *mga2Δ* mutants accumulate Aft1 protein into the vacuole, instead of the nucleus (Jorda et al. 2020), an observation that explains its defects in activation of the iron regulon and growth in iron‐deficient conditions.

In response to iron deficiency, the yeast *S. cerevisiae* inhibits the TORC1 pathway, leading to the repression of bulk translation and the activation of autophagy (Montella‐Manuel et al. 2021; Romero et al. 2019). Autophagy is a highly conserved degradation process that maintains homeostasis by recycling intracellular components, especially in response to stress or nutrient starvation (Nakatogawa et al. 2009; Yin et al. 2016). Autophagy is critical for the recycling of iron during the transition of yeast cells from glycolytic to respiratory growth (Horie et al. 2017). Autophagy and macroautophagy involve the enclosure of proteins, organelles, or even pathogens into double‐membrane vesicles denoted autophagosomes that fuse with the vacuoles. In yeast, the Atg1 kinase complex, composed of Atg1, Atg13, and Atg17 proteins, orchestrates the early steps of autophagosome formation by initiating phagophore nucleation and coordinating downstream autophagy machinery (reviewed in (Mizushima and Komatsu 2011; Nakatogawa et al. 2009; Otto and Thumm 2021)). A specialized form of selective macroautophagy is the cytoplasm‐to‐vacuole targeting (Cvt) pathway, which mediates the transport of specific hydrolases into the vacuolar lumen through the cargo receptor Atg19 (Krick et al. 2008; Lynch‐Day and Klionsky 2010). Microautophagy involves the direct invagination or protrusion of the vacuolar membrane to internalize cellular material (reviewed in Mizushima and Komatsu 2011; Nakatogawa et al. 2009; Otto and Thumm 2021). Micronucleophagy, also known as piecemeal microautophagy of the nucleus (PMN), is a selective form of yeast microautophagy in which portions of the nucleus are pinched off into the vacuole as PMN vesicles and subsequently degraded (reviewed in Mijaljica and Klionsky 2022; Roberts et al. 2003). PMN requires the formation of nucleus‐vacuole junctions (NVJs), which are mediated by a physical interaction between the outer nuclear membrane protein Nvj1 and the vacuolar membrane protein Vac8 (Jeong et al. 2017; Kvam and Goldfarb 2007). Core macroautophagy genes are required for PMN (Krick et al. 2008).

In this study, we discovered that the yeast regulatory factors Aft1 and Cth2 are targeted to the vacuole through PMN when both iron and UFAs levels are limited. Removal of vacuolar targeting does not rescue their regulatory functions, suggesting that this is a route for the elimination of specific non‐functional proteins.

## 2 Materials and Methods

### 2.1 Yeast Strains and Plasmids

The *S. cerevisiae* strains and plasmids employed in this study are detailed in Supporting Information: Supplementary Tables S1 and S2, respectively. Double mutants including *atg1∆mga2∆*, *atg13∆mga2∆*, *atg17∆mga2∆*, *nvj1∆mga2∆*, *vac8∆mga2∆*, *atg19∆mga2∆*, and *vps27∆mga2∆* were generated by integrating an *MGA2* disruption PCR cassette into the corresponding single deletion mutants. The PCR cassette was created using the pFA6a‐His3MX6 plasmid as a template (Longtine et al. 1998) and the primers MGA2‐F1 and MGA2‐R1. Successful disruption of the *MGA2* gene was verified by PCR on genomic DNA using primers TermTEF:135 F and MGA2+193R (Supplementary Table S3). To construct wild‐type and *mga2∆* strains with Nvj1 tagged with GFP at the carboxyl terminus (SPY1343 and SPY1344 strains, respectively), we employed an integrative PCR cassette generated from the pFA6a‐GFP(S65T)‐His3MX6 plasmid (Longtine et al. 1998) with the oligonucleotides NVJ1‐F2 and NVJ1‐R1. The correct integration of GFP was confirmed by PCR using primers NVJ1+212F and promTEF‐74R (Supplementary Table S3).

### 2.2 Yeast Growth Conditions and Transformation

Yeast precultures were grown overnight at 30°C in liquid synthetic complete (SC) medium [0.17% yeast nitrogen base without amino acids and ammonium sulfate (Pronadisa), 0.5% ammonium sulfate (Panreac), 2% glucose (Panreac), and 2 g/L Kaiser drop‐out mix (Formedium)], omitting specific supplements when required. Cultures were reinoculated at an OD_600_ of 0.2 and incubated for 6 h at 190 rpm in either SC medium (+Fe) or SC containing 100 µM of the Fe²⁺‐specific chelator bathophenanthroline disulfonic acid disodium (BPS) (Sigma) to induce iron deficiency (−Fe). To restrict iron bioavailability in solid SC medium (2% agar, Pronadisa), the Fe²⁺‐specific chelator Ferrozine (Sigma) was added at the indicated concentration. For growth assays on solid media, yeast cells were grown to the exponential phase, serially diluted 10‐fold starting from an OD_600_ of 0.1, spotted onto the agar plates, incubated at 30°C for 3 days, and photographed. For liquid growth assays, exponentially growing yeast cells were inoculated into 96‐well plates containing the appropriate medium. Growth was monitored by measuring the OD_600_ every 30 min over a 72 h‐period, after a pre‐shaking of 20 s, using a SpectroStar Nano 96‐well plate reader (BMGLabtech). Yeast cells were transformed by the PEG/SS Carrier DNA/LiAc method (Gietz and Schiestl 2007).

### 2.3 RNA Analyses

Total RNA was extracted, and mRNA expression levels were quantified by RT‐qPCR as previously described (Sanvisens et al. 2014). Briefly, yeast cells were lysed using a Millmix 20 bead beater (Tehtnica) in LETS buffer with acid phenol‐chloroform and glass beads. Supernatants were recovered by phenol‐chloroform extraction. RNA was precipitated, dissolved in RNase‐free Milli‐Q water, and quantified using a NanoDrop spectrophotometer (Thermo Scientific). Subsequently, RNA samples were treated with RNase‐free DNase I (Roche) and reverse‐transcribed with Maxima Reverse Transcriptase (Thermo Scientific) according to the manufacturer's instructions. RT‐qPCR was performed on a Light Cycler 480 II instrument (Roche) using the SYBR Premix *Ex Taq* kit (TaKaRa). Primer sequences used for RT‐qPCR are listed in Supporting Information: Supplementary Table S3. mRNA levels were normalized to *ACT1* expression.

### 2.4 Fluorescence Microscopy

GFP fluorescence and differential interference contrast (DIC) images were acquired using an Eclipse 90i microscope (Nikon Corporation, Japan) equipped with a Nikon DS‐5Mc digital camera. For quantification, over 100 cells from at least three biologically independent experiments were analyzed based on the subcellular localization of GFP‐tagged proteins, categorizing cells according to a predominantly cytoplasmic, nuclear, vacuolar, or NVJ signal. To label the vacuolar membrane, cells were treated with the dye BioTrack 640 Red C2 (FM4‐64) Synaptic (Merck EMD Millipore), as previously reported (Jorda et al. 2020). For nuclear staining, cells were concentrated and incubated with the dye Hoechst 33258 (Invitrogen) at a final concentration of 2 µg/mL for 15 min at 26°C.

### 2.5 Statistical Treatment of Data

Statistical analyses were performed using R (version 4.5.1). Differences between the groups were evaluated by analysis of variance (ANOVA) followed by Tukey's multiple comparison test. Distinct letters positioned above the bars indicate statistically significant differences between groups (*p* < 0.05). Shared letters denote groups that do not differ significantly, while different letters indicate a significant difference between the corresponding means.

## 3 Results

### 3.1 Autophagy Components Contribute to the Vacuolar Accumulation of Aft1 in Iron Starved *mga2Δ* Cells

In yeast, proteins can be directed to the vacuole through different pathways, which share morphological features and depend on overlapping sets of gene products. In order to explore the routes implicated in the mislocalization of Aft1 protein to the vacuole in iron‐deficient *mga2Δ* cells, double mutants lacking *MGA2* and genes implicated in autophagy (*ATG1*, *ATG13*, *ATG17*), cytoplasm‐to‐vacuole targeting (Cvt) (*ATG19*), or endosomal transport of transmembrane proteins to the vacuole (*VPS27*) were constructed. Subsequently, we investigated whether the disruption of any of these pathways would impede the accumulation of GFP‐Aft1 protein into the vacuole of *mga2Δ* cells in the absence of iron.

**Figure 1:**
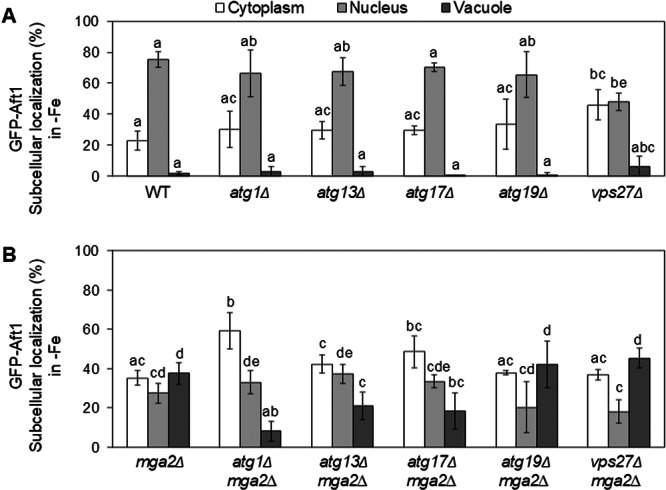
Contribution of different pathways to the vacuolar localization of Aft1 protein in iron‐deficient *mga2Δ* cells. (A) Wild‐type (WT, BY4741), *atg1Δ*, *atg13Δ*, *atg17Δ*, *atg19Δ*, and *vps27Δ*; and (B) *mga2Δ*, *atg1Δmga2Δ*, *atg13Δmga2Δ*, *atg17Δmga2Δ*, *atg19Δmga2Δ*, and *vps27Δmga2Δ* yeast cells expressing GFP‐Aft1 were cultivated for 6 h in iron‐deficient conditions (SC + 100 μM BPS) and analyzed by fluorescence microscopy for subcellular Aft1 localization. At least 100 cells from at least three biologically independent experiments were analyzed, and cells were classified according to a predominantly cytoplasmic, nuclear, or vacuolar signal. Vacuolar membranes were stained with FM4‐64 dye, and nuclei were visualized with Hoechst 33258. Different letters above bars represent statistically significant differences (*p* < 0.05).

First, we observed that the subcellular distribution of GFP‐Aft1 under iron‐deficient conditions was not significantly altered in the single mutants *atg1Δ*, *atg13Δ*, *atg17Δ*, and *atg19Δ* compared with wild‐type cells (Figure 1A). Only the *vps27Δ* mutant showed a mildly altered distribution (Figure 1A). As previously reported (Jorda et al. 2020), the *mga2Δ* mutant displayed a pronounced accumulation of GFP‐Aft1 within the vacuole (Figure 1B). Importantly, deletion of *ATG1* in the *mga2Δ* background markedly reduced the vacuolar accumulation of GFP‐Aft1, restoring levels comparable to those observed in wild‐type cells (Figure 1). The activity of Atg1 is stimulated by its interaction with Atg13 and Atg17 during starvation‐induced autophagy (Mizushima and Komatsu 2011; Nakatogawa et al. 2009; Otto and Thumm 2021). We observed that the accumulation of GFP‐Aft1 into the vacuole in *mga2Δ* cells was also significantly reduced in the *atg13Δmga2Δ* and *atg17Δmga2Δ* mutants, although this reduction was less pronounced than that observed in the *atg1Δmga2Δ* mutant (Figure 1). In contrast to Atg13 and Atg17, Atg1 is also required for vesicle formation in the Cvt pathway. The deletion of the Cvt pathway receptor Atg19 in *mga2Δ* cells had no effect on Aft1 localization, thus excluding the involvement of the Cvt pathway in Aft1 mislocalization (Figure 1). In a similar manner, the deletion of *VPS27*, which is involved in the endosomal pathway for the transport of transmembrane proteins to the vacuole (Bilodeau et al. 2002), did not affect the localization GFP‐Aft1 in *mga2Δ* cells (Figure 1). These results provide substantial evidence to suggest that autophagy plays a pivotal role in the vacuolar accumulation of Aft1 protein in cells exhibiting defects in UFA biosynthesis.

### 3.2 The Piecemeal Microautophagy of the Nucleus Is Activated in Iron‐Deficient *mga2Δ* Cells

**Figure 2:**
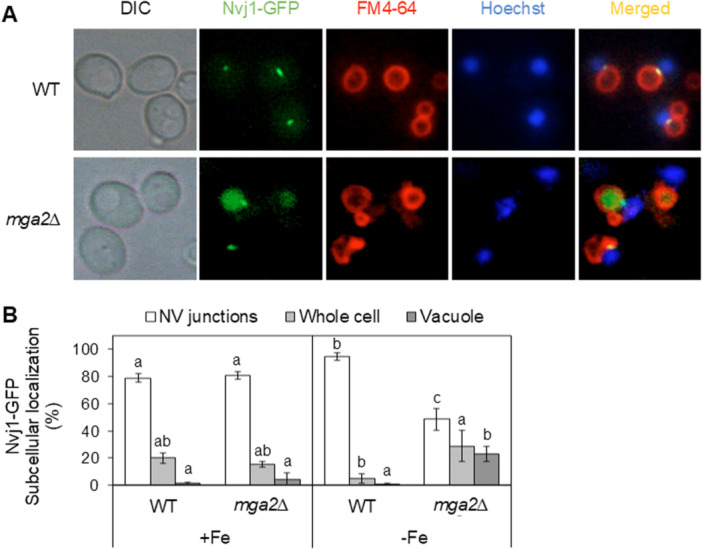
Activation of piecemeal nuclear microautophagy occurs in iron‐deficient *mga2Δ* cells. Wild‐type (WT) and *mga2Δ* yeast cells genomically expressing Nvj1‐GFP protein were cultivated in SC (Panel A, +Fe) and SC + 100 μM BPS (−Fe) for 6 h to exponential growth phase, and analyzed by fluorescence microscopy for subcellular Nvj1 localization. Data were obtained from a minimum of 100 cells across at least three independent biological experiments, and cells were photographed (Panel A) and classified according to a predominantly NV junction, vacuolar, or whole cell localization of the Nvj1 signal (Panel B). Vacuolar membranes and nuclei were visualized with FM4‐64 and Hoechst 33258, respectively. DIC, differential interference contrast. Bars with different letters differ significantly (*p* < 0.05).

Since Aft1 normally accumulates in the nucleus during iron starvation, its mislocalization to the vacuole in *mga2Δ* cells raises the possibility that it is being rerouted from the nucleus. The complex Atg1‐Atg13‐Atg17 is required for the PMN, in which vesicles containing nuclear material are generated and released inside vacuoles (Krick et al. 2008). PMN occurs at NVJs, which are formed through interactions between the outer nuclear membrane protein Nvj1 and the vacuole membrane protein Vac8. These junctions are incorporated into PMN vesicles, resulting in the turnover of Nvj1 (Roberts et al. 2003). In order to investigate PMN activation, genomic *NVJ1* was tagged with GFP in wild‐type and *mga2Δ* cells, and its cellular localization was analyzed under conditions of both iron repletion (+Fe) and depletion (−Fe) using fluorescence microscopy (Figure 2A). In wild‐type cells, Nvj1‐GFP was found to localize to the contact zones between the vacuole (stained with FM4‐64) and the nucleus (stained with Hoechst) in both iron‐replete and iron‐restricted conditions (Figure 2B). This finding is consistent with the presence of Nvj1‐GFP at NVJs. A comparable outcome was achieved for iron‐replete *mga2Δ* cells (Figure 2B). Conversely, in iron‐depleted *mga2Δ* cells, the association of Nvj1‐GFP with NVJs decreased, and a significant accumulation in the vacuole was observed (Figure 2B). The sequestration of Nvj1 into the vacuole suggests that PMN activity is enhanced in *mga2Δ* cells under iron deficiency.

### 3.3 Piecemeal Microautophagy of the Nucleus Promotes the Accumulation of Aft1 Into the Vacuole of Iron‐Deficient *mga2Δ* Cells

**Figure 3:**
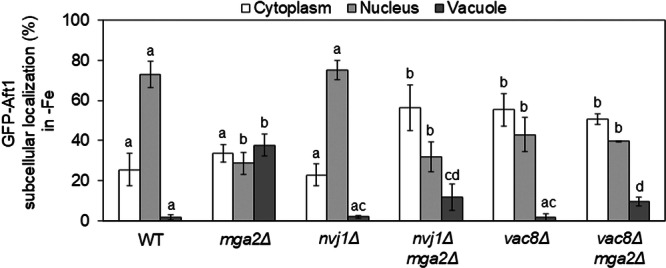
Piecemeal nuclear microautophagy drives Aft1 accumulation to the vacuole under iron deficiency in *mga2Δ* cells. Wild‐type (WT), *mga2Δ*, *nvj1Δ*, *nvj1Δmga2Δ*, *vac8Δ*, and *vac8Δmga2Δ* cells expressing GFP‐Aft1 were cultivated and analyzed in parallel to the strains analyzed in Figure 1. Results for WT and *mga2Δ* have been included to facilitate comparison.

Since PMN is activated in response to iron deficiency in *mga2Δ* cells (Figure 2), we decided to investigate whether PMN is involved in the accumulation of Aft1 in the vacuole (Figure 1). For this purpose, we deleted *NVJ1* and *VAC8* in *mga2Δ* cells, and compared GFP‐Aft1 localization in both single and double mutants as compared to wild‐type and *mga2Δ* cells. As shown in Figure 3, the vacuolar accumulation of GFP‐Aft1 detected in *mga2Δ* cells was reduced in both *nvj1Δmga2Δ* and *vac8Δmga2Δ* mutants, reaching levels similar to those in *atg1Δmga2Δ* cells (Figure 1). As previously observed for *vps27Δ* cells (Figure 1), *vac8Δ* single mutants also displayed a partial mislocalization of GFP‐Aft1 outside the nucleus when compared to wild‐type cells (Figure 3). In sum, these results strongly suggest that PMN is associated with the mislocalization of Aft1 protein to the vacuole in iron‐depleted *mga2Δ* cells.

### 3.4 Prevention of Aft1 Vacuolar Accumulation in *mga2Δ* Cells Fails to Restore Its Role in Transcriptional Activation of the Iron Regulon and Growth in Iron Deficiency

**Figure 4:**
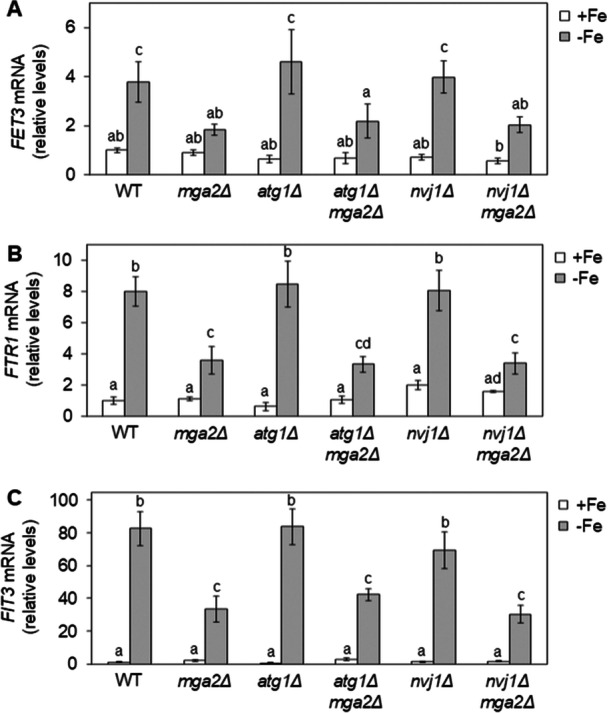
Aft1 vacuolar exclusion in *mga2Δ* cells does not restore iron regulon activation. Wild‐type (WT), *mga2Δ*, *atg1Δ*, *atg1Δmga2Δ*, *nvj1Δ*, and *nvj1Δmga2Δ* cells were cultivated in SC (+Fe) and SC + 100 μM BPS (−Fe) for 6 h, and the expression of *FET3* (A), *FTR1* (B), and *FIT3* (C) mRNAs was analyzed by RT‐qPCR. Values were normalized to *ACT1* and made relative to WT cells in +Fe conditions. The average and standard deviation of at least three biologically independent experiments are represented. Different letters above bars represent statistically significant differences (*p* < 0.05).

In earlier studies, we demonstrated that Aft1 mislocalization in *mga2Δ* cells is associated with impaired activation of the iron regulon and compromised cellular growth under iron‐deficient conditions (Jorda et al. 2020). Consequently, we decided to assess whether preventing Aft1 vacuolar accumulation in iron‐depleted *mga2Δ* cells could rescue these phenotypes. First, we examined iron regulon activation by cultivating wild‐type, *mga2Δ*, *atg1Δ*, *nvj1Δ*, *atg1Δmga2Δ*, and *nvj1Δmga2Δ* cells under both iron‐sufficient and iron‐deficient conditions, followed by RT‐qPCR quantification of mRNA levels for several iron regulon genes. As expected, the transcript levels of *FET3*, *FTR1*, and *FIT3* increased in wild‐type cells in response to iron deprivation, whereas this up‐regulation was markedly attenuated in *mga2Δ* mutants (Figure 4). The deletion of either *ATG1* or *NVJ1* alone did not affect the iron regulon expression in comparison to wild‐type cells (Figure 4). Importantly, the deletion of either *ATG1* or *NVJ1* in *mga2Δ* cells failed to restore the activation of the iron regulon in response to iron deficiency (Figure 4). While disruption of the PMN machinery reduced Aft1 accumulation into the vacuole, the resulting cytosolic/nuclear localization pattern remained inconsistent with the nuclear enrichment characteristic of active Aft1 in iron‐depleted wild‐type cells (Figures 1 and 3). These results indicate that the removal of PMN prevents Aft1 accumulation into the vacuole; however, this is not sufficient to restore proper nuclear retention of Aft1 and iron regulon activation in response to iron deficiency.

**Figure 5:**
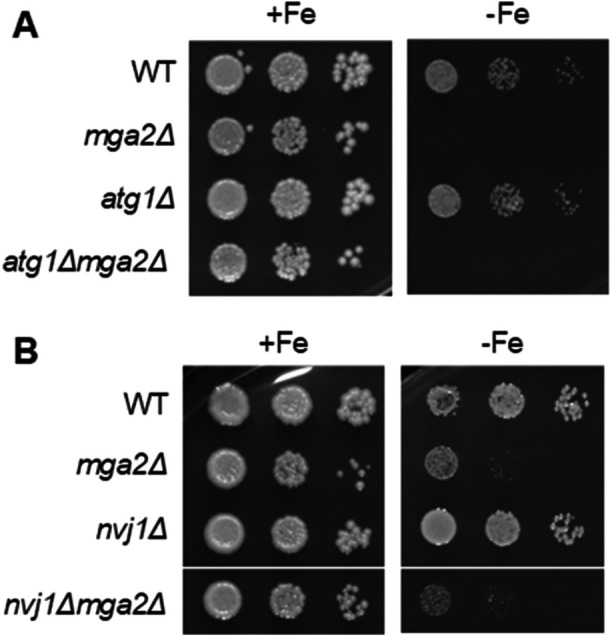
Aft1 vacuolar exclusion in *mga2Δ* cells is insufficient to recover growth under iron‐deficient conditions**.** Wild‐type (WT), *mga2Δ*, *atg1Δ*, *atg1Δmga2Δ*, *nvj1Δ*, and *nvj1Δmga2Δ* cells were cultivated to exponential growth phase and spotted in SC (+Fe) and SC + 500 μM Ferrozine (−Fe) in 10‐fold serial dilutions. In all cases, plates were incubated for 3–4 days at 30°C and then photographed. A representative experiment is shown.

Secondly, the effect of removing the PMN pathway on the yeast fitness was evaluated by testing their growth in both solid and liquid media with low iron bioavailability. No remarkable variations in growth patterns were observed among the strains under iron‐sufficient conditions (Figure 5). In conditions where iron bioavailability was limited, the growth rate of *atg1Δ* and *nvj1Δ* cells exhibited a comparable trend to that observed in wild‐type cells (Figure 5). As previously reported (Romero et al. 2018), *mga2Δ* cells exhibited a substantial growth defect in conditions of low iron. Notably, the additional deletion of *ATG1* or *NVJ1* in *mga2Δ* did not rescue normal growth (Figure 5). Collectively, these findings suggest that, while disrupting the PMN machinery prevents Aft1 accumulation in the vacuole of cells lacking *MGA2* under iron deficiency, it does not restore the normal subcellular distribution and function of the Aft1 transcription factor and, consequently, does not rescue growth under iron‐deficient conditions.

### 3.5 Cth2 Protein, but Not Other Nucleocytoplasmic Shuttling Proteins, Accumulates in the Vacuole of Iron‐Deficient Cells Defective in Unsaturated Fatty Acid Biosynthesis

**Figure 6:**
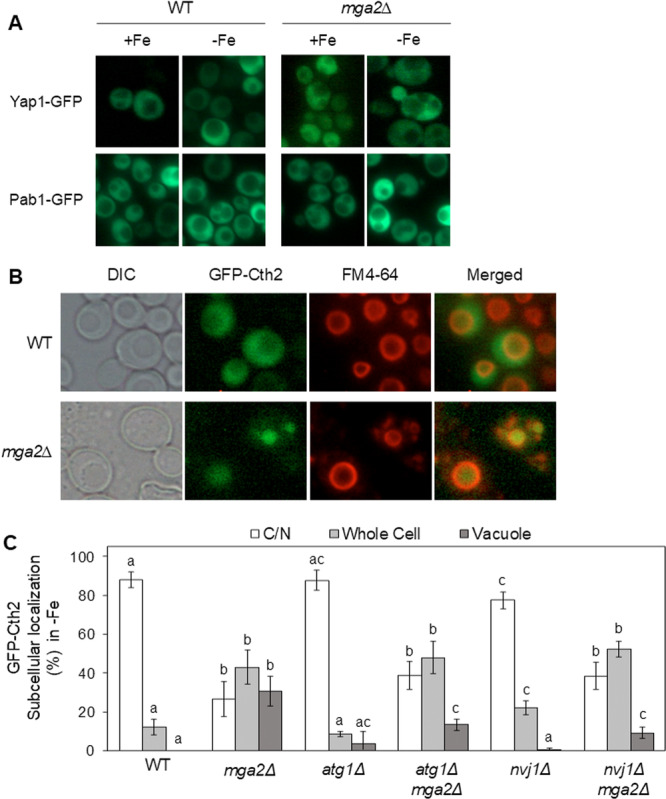
Cth2 localizes to the vacuole in iron‐deficient cells defective in unsaturated fatty acid production. (A) Wild‐type (WT) and *mga2Δ* cells expressing Yap1‐GFP or Pab1‐GFP proteins were cultivated in SC (+Fe) and SC + 100 μM BPS (−Fe) for 6 h to reach exponential growth phase, and analyzed by fluorescence microscopy. (B) Wild‐type and *mga2Δ* cells expressing GFP‐Cth2 protein were cultivated and analyzed as in panel A. Vacuolar membranes were visualized with FM4‐64. Representative images are shown. (C) Wild‐type, *mga2Δ*, *atg1Δ*, *atg1Δmga2Δ*, *nvj1Δ*, and *nvj1Δmga2Δ* cells expressing GFP‐Cth2 cells were cultivated and analyzed as in panel A and classified according to a predominantly vacuolar, cytoplasmic/nuclear (C/N), or whole cell localization. Data were collected from at least 100 cells in a minimum of three independent biological replicates. Bars with different letters differ significantly (*p* < 0.05).

In order to investigate whether other proteins that constantly transit between the nucleus and the cytoplasm are similarly mislocalized to the vacuole in *mga2∆* cells, the subcellular localization of other GFP‐tagged shuttling proteins was assessed in wild‐type (WT) and *mga2Δ* cells cultivated in both iron‐sufficient and iron‐deficient conditions by fluorescence microscopy. We observed that the transcription factor Yap1, which is essential for the oxidative stress response (reviewed in Rodrigues‐Pousada et al. 2019), and the poly(A)‐binding protein Pab1, which is involved in regulating the length of poly(A) tails on nuclear mRNA transcripts (reviewed in Brambilla et al. 2019), were equally distributed in wild‐type and *mga2Δ* cells under both iron replete and iron depleted conditions (Figure 6A). Conversely, the shuttling mRNA‐binding protein Cth2, whose expression is induced by Aft1 in the response to iron depletion (Puig et al. 2005; Rutherford et al. 2003), exhibited significant mislocalization in *mga2∆* cells in comparison to wild‐type cells under iron‐limiting conditions (Figure 6B). The co‐localization of Cth2 with the vacuolar dye FM4‐64, as well as the subsequent quantification of its subcellular localization patterns, was found to confirm the accumulation of Cth2 into the vacuole of iron‐deficient *mga2Δ* cells (Figure 6B,C, WT and *mga2Δ* cells). We also noted that a considerable proportion of *mga2Δ* cells displayed a diffuse distribution of the GFP‐Cth2 protein, encompassing both the cytoplasm and the vacuole (Figure 6C, whole cell). Collectively, these findings suggest that vacuolar mislocalization in response to iron and UFAs defects is not a universal phenomenon affecting all nucleocytoplasmic shuttling proteins. Rather, it encompasses two iron‐responsive proteins, namely Aft1 and Cth2. This observation indicates a particular disturbance in the localization of iron‐related proteins in *mga2∆* cells.

### 3.6 The Cth2‐Mediated Down‐Regulation of Target Transcripts in Response to Iron Deficiency Is Impaired in *mga2Δ* Cells

**Figure 7:**
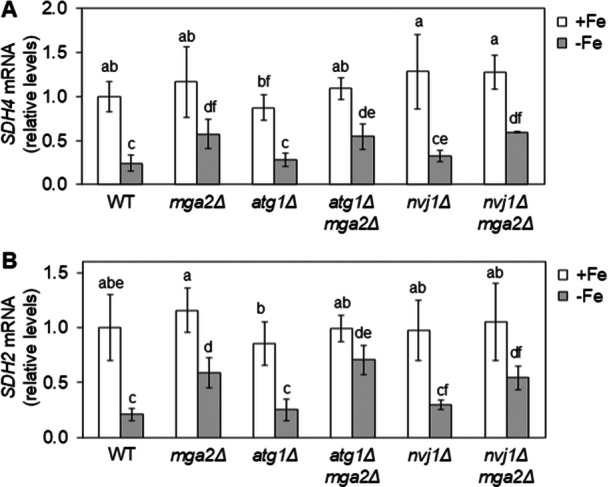
The Cth2‑dependent down‑regulation of target mRNAs in response to iron deficiency is compromised in *mga2Δ* cells. Wild‐type (WT), *mga2Δ*, *atg1Δ*, *atg1Δmga2Δ*, *nvj1Δ*, and *nvj1Δmga2Δ* cells were cultivated in SC (+Fe) and SC + 100 μM BPS (−Fe) for 6 h, and the expression of *SDH4* (A) and *SDH2* (B) mRNAs were analyzed by RT‐qPCR. Data were normalized against *PGK1* transcript levels and presented as fold change relative to WT cells in +Fe medium. Data represent the mean ± standard deviation from two to six independent biological replicates. Distinct letters above bars indicate statistically significant differences (*p* < 0.05).

Cth2 promotes the degradation of many mRNAs, including succinate dehydrogenase subunits encoded by *SDH2* and *SDH4* in response to iron deficiency (Puig et al. 2005; Pedro‐Segura et al. 2008). To evaluate whether Cth2 functionality was affected in the *mga2Δ* mutant, we determined the transcript levels of *SDH2* and *SDH4* under +Fe and −Fe. While iron depletion prominently reduced both transcript levels in wild‐type cells, the drop was significantly attenuated in *mga2Δ* mutants (Figure 7, WT and *mga2Δ* cells), strongly suggesting a defect in Cth2 function.

### 3.7 Deletion of Autophagy Factors Does Not Rescue Cth2 Localization and Regulatory Functions in Iron‐Deficient *mga2Δ* Cells

Since the functional defect of Cth2 in *mga2Δ* is probably due to its mislocalization to the vacuole, we ascertain whether deletion of autophagy factors could restore Cth2 nucleo‐cytoplasmic shuttling and targeted mRNA decay function. Firstly, the subcellular localization of GFP‐Cth2 was determined in *atg1Δmga2Δ* and *nvj1Δmga2Δ* double mutants in comparison to wild‐type, *mga2Δ*, *atg1Δ*, and *nvj1Δ* cells under both iron‐replete and iron‐deplete conditions. As shown in Figure 6C, the nucleo‐cytoplasmic localization of Cth2 was not significantly altered in *atg1Δ* and *nvj1Δ* mutants. Importantly, deletion of *ATG1* or *NVJ1* in *mga2Δ* mutants significantly decreased the accumulation of GFP‐Cth2 into the vacuole (Figure 6C), consistently with PMN being implicated in its vacuolar mislocalization. However, as was previously observed for Aft1, the removal of PMN did not fully rescue the nucleo‐cytoplasmic localization of Cth2. In this case, a marked diffuse distribution was exhibited by many *atg1Δmga2Δ* and *nvj1Δmga2Δ* cells throughout the whole cell when compared to wild‐type cells (Figure 6C). When mRNA down‐regulation was examined, we observed that *atg1Δmga2Δ* and *nvj1Δmga2Δ* cells were unable to regain the capacity of wild‐type cells to down‐regulate *SDH2* and *SDH4* mRNA levels in response to iron starvation (Figure 7). Collectively, these results indicate that PMN triggers the accumulation of Cth2 protein into the vacuole in iron‐deficient *mga2Δ* cells, but disrupting this pathway does not rescue Cth2 function in mRNA decay despite partially recovering its nucleo‐cytoplasmic localization.

## 4 Discussion

In this study, we show that the iron‐regulated proteins Aft1 and Cth2 accumulate in the vacuole when yeast cells experience simultaneous UFA and iron limitation. Although inhibition of autophagy‐ and PMN‑related pathways prevents their mislocalization, it neither restores their regulatory functions nor improves growth under low‑iron conditions. These results suggest that PMN primarily eliminates non‑functional iron‑regulated proteins under dual nutrient stress, acting as a quality‑control mechanism rather than enabling functional recovery.

Defects in diverse lipid pathways, including UFAs, sterols, and sphingolipids, converge to promote vacuolar mislocalization of the iron‐responsive transcription factor Aft1 in *S. cerevisiae* under iron‐starved conditions. In *mga2Δ* mutants, which exhibit defects in UFA synthesis, Aft1 fails to accumulate in the nucleus and is instead redirected to the vacuole, impairing iron regulon activation, a phenotype that is reversed by exogenous UFA or *OLE1* expression (Jorda et al. 2020). Similarly, yeast strains with disrupted ergosterol biosynthesis mislocalize Aft1 to the vacuole and exhibit compromised growth in low iron, underscoring sterol requirement for correct Aft1 localization (Jorda et al. 2021). Furthermore, inhibition of sphingolipid production or perturbation of TORC2/Ypk1 activity, which is critical for sphingolipid synthesis, impairs nuclear translocation of Aft1, leading to its cytoplasmic or vacuolar redistribution under iron depletion (Montella‐Manuel et al. 2023). These findings reveal that multiple lipid classes are necessary to ensure proper Aft1 targeting and iron homeostasis, and that defects in any of these lipid biosynthetic routes promote vacuolar sequestration of Aft1, effectively decoupling iron sensing from downstream transcriptional responses.

In order to investigate the molecular mechanisms responsible for Aft1 mislocalization upon defects in lipid biosynthesis, the present study has focused on cells defective in UFAs, but the investigation has been extended to other nucleo‐cytoplasmic shuttling proteins. The selective vacuolar accumulation of Aft1 and Cth2 proteins in iron‐deficient *mga2Δ* cells, in contrast to the unaffected localization of other nucleo‐cytoplasmic shuttling proteins such as Yap1 and Pab1, indicates that there is not a general collapse of nucleo‐cytoplasmic transport or protein quality control mechanisms but rather suggests a targeted disruption affecting iron homeostasis‐related pathways. However, whether this effect is specific to iron‐regulated proteins remains unresolved, as only a limited number of shuttling factors have been tested. A broader analysis encompassing a wider range of nuclear and cytoplasmic proteins would be necessary to determine the true extent of this selectivity. Moreover, the mechanism by which the cell distinguishes Aft1 and Cth2 from other unaffected shuttling proteins under these conditions remains unknown. It is unclear whether specific features of their structure, post‐translational modifications, or interaction partners underlie their selective targeting to the vacuole, raising intriguing questions about the molecular cues driving this differential handling.

Previous studies have shown that autophagy is required for the proper transition of yeast cells from fermentative to respiratory growth (Horie et al. 2017). Notably, supplementation with iron, an essential cofactor for respiratory metabolism, rescues the growth defects of autophagy mutants during the diauxic shift, suggesting that autophagy contributes to iron recycling, likely through vacuolar transporter systems or the degradation of iron‐containing substrates (Horie et al. 2017). More recently, additional findings in *S. cerevisiae* have demonstrated that iron limitation inhibits the TORC1 pathway and thereby induces bulk autophagy (Montella‐Manuel et al. 2021; Romero et al. 2019). Although TORC1 inhibition by rapamycin promotes PMN in *S. cerevisiae* (Roberts et al. 2003), we do not detect PMN induction after 6 h of iron depletion. A plausible explanation is that PMN may require more severe iron starvation to be fully activated. In any case, our results show that UFA depletion caused by *MGA2* deletion triggers PMN under iron‐deficient conditions.

In response to various stress conditions, PMN preserves nuclear homeostasis by selectively engulfing portions of the nucleus to degrade and recycle non‐essential, undesirable, or damaged nuclear components in the vacuole (Mijaljica and Klionsky 2022; Roberts et al. 2003). However, the regulation of this quality control mechanism under lipid‐ or iron‐deficient conditions remains poorly understood. NV junctions are known to recruit proteins involved in lipid metabolism, including Tsc13 and Osh1, which participate in fatty acid and sterol metabolic pathways, respectively (Kvam and Goldfarb 2007). Although *mga2Δ* mutants exhibit severe defects in UFA synthesis, we do not observe enhanced PMN activity or altered protein localization under iron‐replete conditions. Only when both UFA and iron availability are limited do the two nucleo‐cytoplasmic shuttling proteins Aft1 and Cth2 become substantially redirected from the nucleus to the vacuole. We speculate that both proteins are captured within the nucleus because their nuclear localization is essential for their regulatory functions in gene expression. Aft1 binds the promoters of its iron‐regulon target genes in the nucleus (Yamaguchi‐Iwai et al. 1996), whereas Cth2 post‐transcriptional regulator, interacts cotranscriptionally with ARE‐containing transcripts and associated protein factors during its transit through the nucleus (Perea‐Garcia et al. 2020; Prouteau et al. 2008; Vergara et al. 2011).

While PMN‐mediated autophagy contributes to the vacuolar accumulation of Aft1 and Cth2 proteins, blocking this pathway does not fully restore their proper subcellular distribution or function. This observation indicates that the primary defect in *mga2Δ* cells not only alters protein trafficking but also affects upstream processes essential for the regulatory function of Aft1 and Cth2. In *S. cerevisiae*, PMN has been proposed to function as a quality‐control system that removes damaged or unnecessary nuclear material (Kvam and Goldfarb 2007). The PMN‐mediated delivery of Aft1 and Cth2 to the vacuole may therefore represent a mechanism for eliminating non‐functional iron‐regulatory proteins when both UFA and iron availability are compromised. Overall, these findings uncover a previously unappreciated connection between lipid metabolism and iron signaling that specifically impairs the localization and activity of central iron‐regulatory factors, and they open new avenues for investigating the mechanistic specificity underlying this process.

## Author Contributions

**Sergi Puig:** conceptualization, resources, writing – original draft, supervision, project administration, funding acquisition. **Tania Jordá:** investigation, methodology, formal analysis, writing – original draft, visualization.

## Ethics Statement

The authors have nothing to report.

## Conflicts of Interest

The authors declare no conflicts of interest.

## Supporting information


**Supplementary Table S1.** List of yeast strains used in this study. **Supplementary Table S2.** List of plasmids used in this study. **Supplementary Table S3.** Oligonucleotides used for RT‐qPCR in this work.

## Data Availability

Supporting data for this study are provided in the article and its supplementary files. The primary datasets used in the preparation of this manuscript and their analyses are openly available in Digital CSIC (http://hdl.handle.net/10261/413265).
