# Supplementary material for: Nuclear Microautophagy Drives Vacuolar Targeting of Yeast Iron‐Regulated Proteins During Lipid and Iron Limitation
Source: Microbiologyopen. 2026 Apr 16;15(2):e70278. doi: 10.1002/mbo3.70278 (PMC13084708; doi:10.1002/mbo3.70278)
Supplement: Supplementary file 1 — Supplementary Table S1. List of yeast strains used in this study. Supplementary Table S2. List of plasmids used in this study. Supplementary Table S3. Oligonucleotides used for RT‐qPCR in this work. [file MBO3-15-e70278-s001.docx]

**Supplementary Tables**

**Supplementary Table S1.** List of yeast strains used in this study.

| Strain | Description | Source |
| --- | --- | --- |
| BY4741 | *MATa his3Δ1 leu2Δ0 met15Δ0 ura3Δ0* | Invitrogen |
| SPY824 | BY4741 *mga2::KanMX4* | Invitrogen |
| SPY1168 | BY4741 *atg1::KanMX4* | Invitrogen |
| SPY1214 | BY4741 *atg13::KanMX4* | Invitrogen |
| SPY1216 | BY4741 *atg17::KanMX4* | Invitrogen |
| SPY1169 | BY4741 atg19*::KanMX4* | Invitrogen |
| SPY1167 | BY4741 *vps27::KanMX4* | Invitrogen |
| SPY1237 | BY4741 *nvj1::KanMX4* | Invitrogen |
| SPY1310 | BY4741 *vac8::KanMX4* | Invitrogen |
| SPY1202 | BY4741 *atg1::KanMX4 mga2::HIS3MX6* | This study |
| SPY1215 | BY4741 *atg13::KanMX4 mga2::HIS3MX6* | This study |
| SPY1217 | BY4741 *atg17::KanMX4 mga2::HIS3MX6* | This study |
| SPY1203 | BY4741 atg19*::KanMX4 mga2::HIS3MX6* | This study |
| SPY1201 | BY4741 *vps27::KanMX4 mga2::HIS3MX6* | This study |
| SPY1314 | BY4741 *nvj1::KanMX4 mga2::HIS3MX6* | This study |
| SPY1311 | BY4741 *vac8::KanMX4 mga2::HIS3MX6* | This study |
| SPY1343 | BY4741 *NVJ1-GFP::HIS3MX6* | This study |
| SPY1344 | BY4741 *NVJ1-GFP::HIS3MX6 mga2::KanMX4* | This study |

**Supplementary Table S2. List of plasmids used in this study.**

| Plasmid | Description | Source |
| --- | --- | --- |
| pAC520 | *LEU2* YAP1-GFP | (Jones et al., 2000) |
| pCMH035 | *URA3* PAB1-GFP | (Dunn, Hammell, Hodge, & Cole, 2005) |
| pSP412 | *URA3* GFP-CTH2 | (Vergara, Puig, & Thiele, 2011) |
| JK1346 | *URA3* GFP-AFT1 | (Crisp et al., 2003) |
| pFA6a-His3MX6 | *P_TEF_-his5^+^-T_TEF_* | (Longtine et al., 1998) |
| pFA6a-GFP(S65T)-His3MX6 | *GFP(S65T)-T_ADH_-P_TEF_-his5^+^-T_TEF_* | (Longtine et al., 1998) |

**Supplementary Table S3. Oligonucleotides used for RT-qPCR in this work.**

| ***Name*** | ***Sequence (from 5’ to 3’)*** |
| --- | --- |
| FET3-qPCR-F | TGACCGTTTTGTCTTCAGGT |
| FET3-qPCR-R | CTCCACGATTTCATCCTTCTC |
| FTR1-qPCR-F | GGTCACTTGCCTTTCACCAA |
| FTR1-qPCR-R | TTGCTCTTCCGTCAACTCCT |
| FIT3-qPCR-F | CATCCTCTAGCACCGCTGAA |
| FIT3-qPCR-R | CAATAACATGACGGCAGCAA |
| SDH2-qPCR-F | CGAAGAAGGGTATGGCTACTG |
| SDH2-qPCR-R | CACTTGGCTCGTCTGGATT |
| SDH4-qPCR-F | GCACTCCCAATGATGCCTAC |
| SDH4-qPCR-R | AATGGAACGACGGACAAGG |
| MGA2-F1 | CACTTATTGAAGGTCATTTTGGCGAACAGAACATTTCGTTCGGATCCCCGGGTTAATTAA |
| MGA2-R1 | TTCTCTTCTTTTAAGTGTACTGTCTTTTCATTATACACACGAATTCGAGCTCGTTTAAAC |
| TermTEF:135F | CGACATCATCTGCCCAGAT |
| MGA2+193R | CGATAGACATTGCGCTCGTGTT |
| NVJ1-F2 | AGTGAACACTGAACAAGCATACTCTCAACCATTTAGATACCGGATCCCCGGGTTAATTAA |
| NVJ1-R1 | TCGTTGTAAGTGACGATGATAACCGAGATGACGGAAATATGAATTCGAGCTCGTTTAAAC |
| NVJ1-212F | TGCACAATGGGCTCCTCTTTG |
| promTEF-74R | GGGCGACAGTCACATCAT |
|  |  |

**REFERENCES**

Crisp, R. J., Pollington, A., Galea, C., Jaron, S., Yamaguchi-Iwai, Y., & Kaplan, J. (2003). Inhibition of heme biosynthesis prevents transcription of iron uptake genes in yeast. *J Biol Chem, 278*(46), 45499-45506.

Dunn, E. F., Hammell, C. M., Hodge, C. A., & Cole, C. N. (2005). Yeast poly(A)-binding protein, Pab1, and PAN, a poly(A) nuclease complex recruited by Pab1, connect mRNA biogenesis to export. *Genes Dev, 19*(1), 90-103. doi:10.1101/gad.1267005

Jones, A. L., Quimby, B. B., Hood, J. K., Ferrigno, P., Keshava, P. H., Silver, P. A., & Corbett, A. H. (2000). SAC3 may link nuclear protein export to cell cycle progression. *Proc Natl Acad Sci U S A, 97*(7), 3224-3229. doi:10.1073/pnas.97.7.3224

Longtine, M. S., McKenzie, A., 3rd, Demarini, D. J., Shah, N. G., Wach, A., Brachat, A., . . . Pringle, J. R. (1998). Additional modules for versatile and economical PCR-based gene deletion and modification in Saccharomyces cerevisiae. *Yeast, 14*(10), 953-961.

Vergara, S. V., Puig, S., & Thiele, D. J. (2011). Early recruitment of AU-rich element-containing mRNAs determines their cytosolic fate during iron deficiency. *Mol Cell Biol, 31*(3), 417-429. doi:10.1128/MCB.00754-10
